# Supplementary figures and images for: The Complete Mitochondrial Genome of the Deep-Dwelling Goby Suruga fundicola (Teleostei, Gobiidae) Reveals Evidence of Recombination in the Control Region
Source: Int J Mol Sci. 2025 Aug 27;26(17):8317. doi: 10.3390/ijms26178317 (PMC12428434; doi:10.3390/ijms26178317)

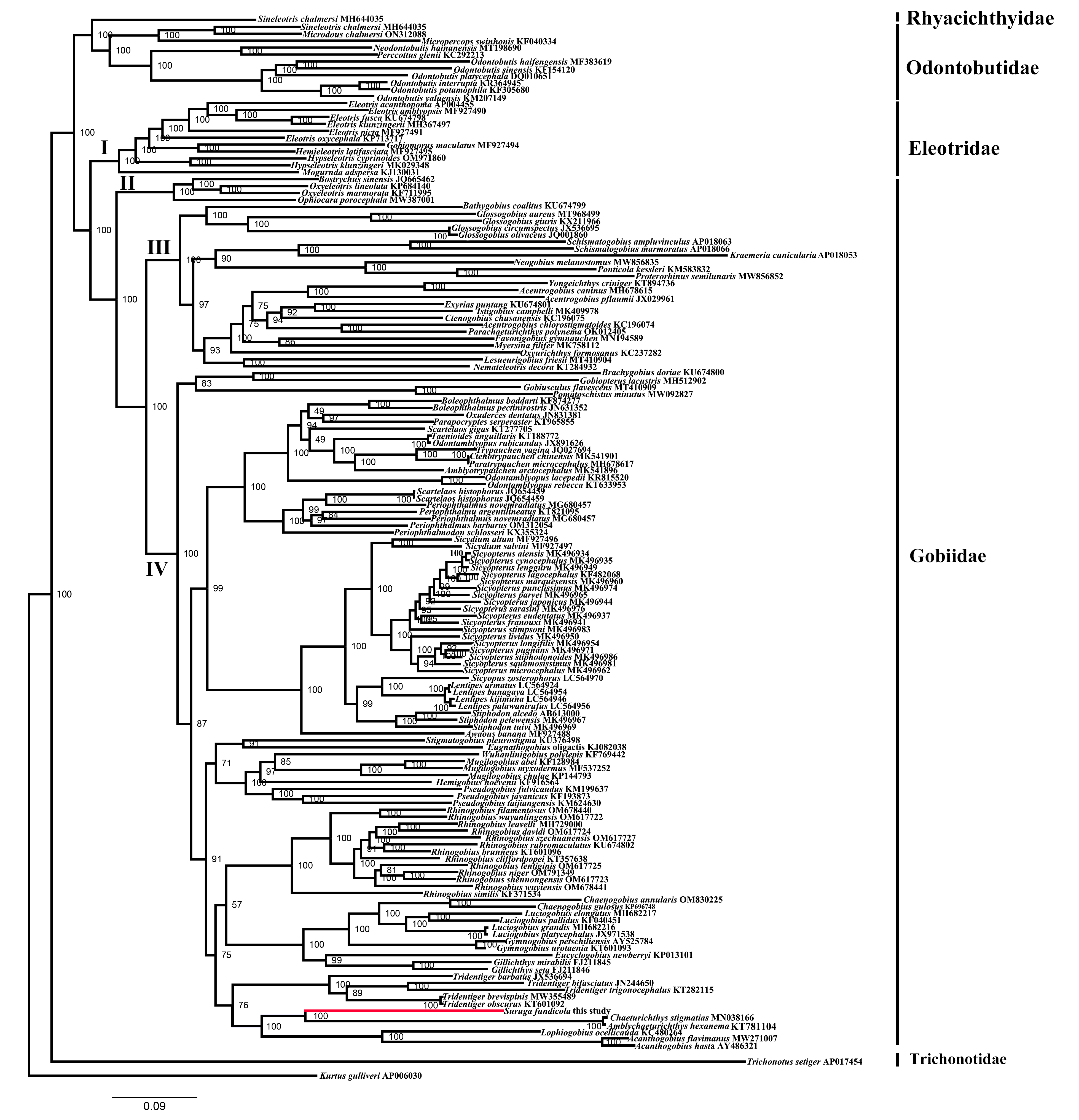

Supplement: Supplementary file 1 [file ijms-26-08317-s001.zip › ijms-3784074-12-supplementary/Supplementary Figure S2. Phylogenetic tree constructed by IQ methods.tif]

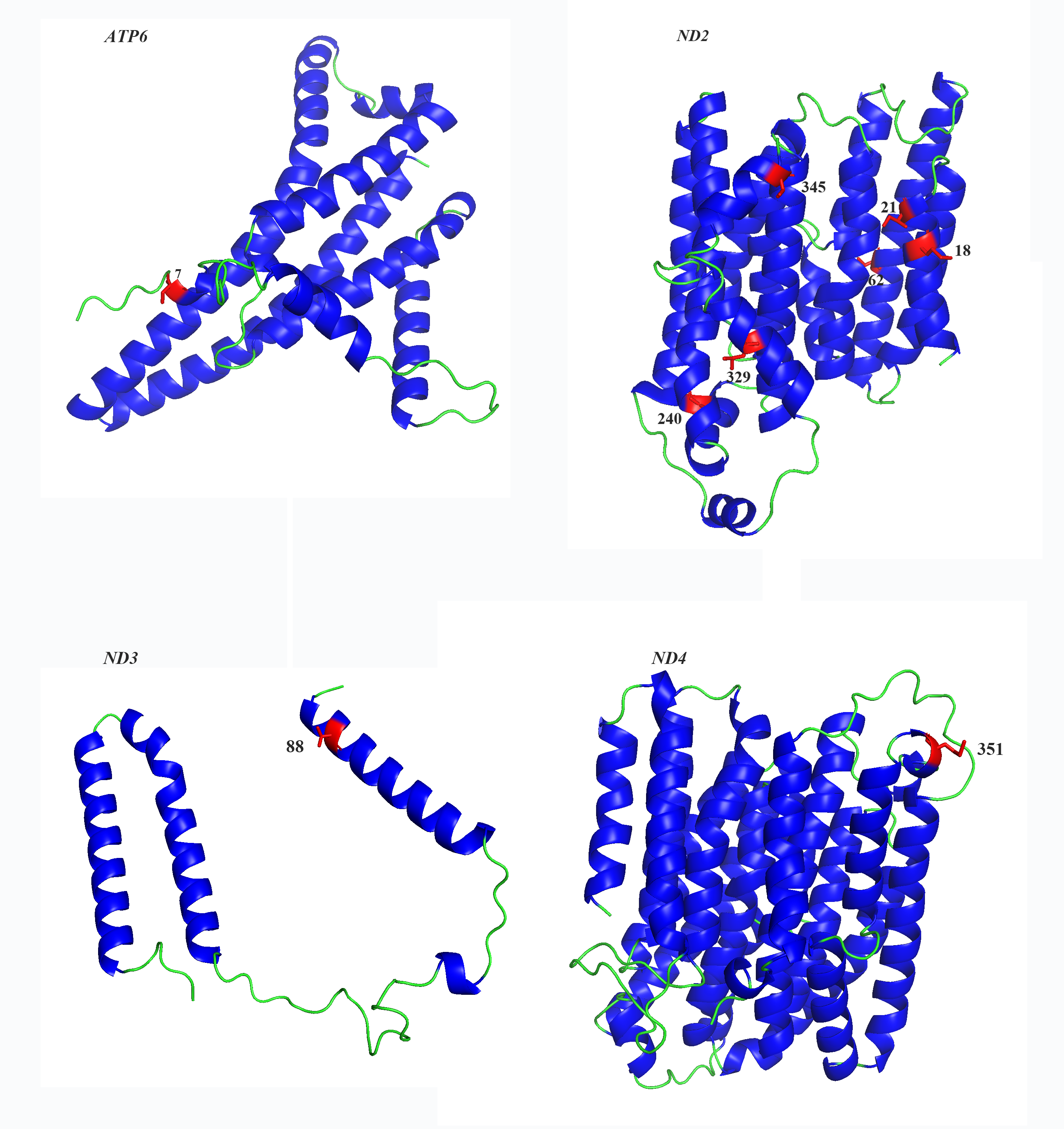

Supplement: Supplementary file 1 [file ijms-26-08317-s001.zip › ijms-3784074-12-supplementary/Supplementary Figure S3. Positive selection sites.tif]
